# Using a qualitative sub-study to inform the design and delivery of randomised controlled trials on medicinal cannabis for symptom relief in patients with advanced cancer

**DOI:** 10.1186/s13063-022-06691-1

**Published:** 2022-09-05

**Authors:** Rebecca E. Olson, Alexandra Smith, Georgie Huggett, Phillip Good, Morgan Dudley, Janet Hardy

**Affiliations:** 1 School of Social Science, The University of Queensland, Michie Building #9, St Lucia, QLD 4072 Australia; 2 Department of Palliative and Supportive Care Mater Health Services, Mater Research-University of Queensland, Raymond Terrace, South Brisbane, QLD Australia; 3 Department of Palliative Care, St. Vincent’s Private Hospital Brisbane, 411 Main Street, Kangaroo Point, QLD Australia

**Keywords:** RCT, Palliative care, Medicinal cannabis, Australia, Qualitative, Recruitment, Patient and public

## Abstract

**Background:**

Recruitment for randomised controlled trials in palliative care can be challenging; disease progression and terminal illness underpin high rates of attrition. Research into participant decision-making in medicinal cannabis randomised controlled trials (RCTs) is very limited. Nesting qualitative sub-studies within RCTs can identify further challenges to participation, informing revisions to study designs and recruitment practices. This paper reports on findings from a qualitative sub-study supporting RCTs of medicinal cannabis for symptom burden relief in patients with advanced cancer in one Australian city.

**Methods:**

Semi-structured qualitative interviews were conducted with 48 patients with advanced cancer, eligible to participate in a medicinal cannabis RCT (*n*=28 who consented to participate in an RCT; *n*=20 who declined). An iterative and abductive approach to thematic analysis and data collection fostered exploration of barriers and enablers to participation.

**Results:**

Key enablers included participants’ enthusiasm and expectations of medicinal cannabis as beneficial (to themselves and future patients) for symptom management, especially after exhausting currently approved options, and a safer alternative to opioids. Some believed medicinal cannabis to have anti-cancer effects. Barriers to participation were the logistical challenges of participating (especially due to driving restrictions and fatigue), reluctance to interfere with an existing care plan, cost, and concerns about receiving the placebo and the uncertainty of the benefit. Some declined due to concerns about side-effects or a desire to continue accessing cannabis independent of the study.

**Conclusions:**

The findings support revisions to subsequent medicinal cannabis RCT study designs, namely, omitting a requirement that participants attend weekly hospital appointments. These findings highlight the value of embedding qualitative sub-studies into RCTs. While some challenges to RCT recruitment are universal, others are context (population, intervention, location) specific. A barrier to participation found in research conducted elsewhere—stigma—was not identified in the current study. Thus, findings have important implications for those undertaking RCTs in the rapidly developing context of medical cannabis.

## Background

Maximising recruitment and minimising attrition are both important for drawing valid conclusions from randomised controlled trials (RCTs) [1]. An estimated 50% of RCTs fail to meet recruitment targets [2]. Poor recruitment prolongs study timelines and increases costs [3, 4]. These setbacks are particularly problematic in palliative medicine clinical trials, which tend to have higher rates of attrition [5].

Key challenges to RCT recruitment identified by systematic reviews include overestimation of eligible participants, poor trial communication and understanding, perceived burden for participants and recruiting clinicians, prejudice related to trial effectiveness, and influence from trusted others on patient decision-making [6–8]. As personal benefit along with benefit to others is key deciding factors, increasing awareness of the health problem, the possible benefits of the intervention being trialled, and engaging eligible participants in the trial process are common strategies for improving recruitment [7, 9–11]. Furthermore, given their centrality to trial communication, training recruiters is another strategy for improving trial recruitment [12].

Although some of these challenges are common to all RCTs, specific trials can also have their own unique challenges [13, 14]. Recruiting patients with advanced cancer within palliative care, for instance, poses significant and distinct challenges. Three central barriers to recruitment have been raised [5]: (1) concern for the patient’s health and future (e.g. frailty, uncertain prognosis); (2) gatekeeping—clinicians and informal carers shielding a patient from the false hope or burden of a clinical trial; and (3) ethical concerns about clinical equipoise, vulnerability, and capacity to consent. As a consequence, older and/or palliative care patients tend to be under-represented in clinical trials more generally [15–19], despite research showing many of these patients’ and family members’ desire to participate in RCTs [20].

Despite these challenges, there has been developing interest in clinical trials involving medicinal cannabis and consideration of its potential use in older patient populations and palliative care contexts [21–26]. Although most Australians approve of medical cannabis clinical trials and medicinal cannabis use in oncology [27, 28], given the history of cannabis as an illicit substance, this may not translate to direct interest in RCT participation. Available research from North America, Israel, and Europe found stigma, risk, and access pathways to be primary concerns around medical cannabis [29–36]. Research in the Australian setting, a unique social, regulatory, and clinical context, is sparse [37]. Where it exists, studies are largely survey-based, assessing general attitudes to medicinal cannabis [22, 38–41]. Research into patients’ perceptions of medicinal cannabis and trial participation decision-making is even more limited—in large part because clinical trials of medicinal cannabis in Australia have only recently emerged in significant numbers [42, 43]. Available studies show few Australian participants hold concerns related to adverse effects; a minority believe cannabis might cure cancer [40, 41, 43, 44]. Overall, however, little is known about Australian patients’ perceptions of medicinal cannabis and even less is known about enablers and barriers to recruiting patients with advanced cancer to medicinal cannabis RCTs [26, 35].

International guidelines advocate for the use of qualitative sub-studies within RCTs to identify and address recruitment challenges, specific to local contexts and cultures [45–47]. Qualitative research can improve the potential viability and depth of RCT findings [13, 48]. Moreover, qualitative sub-studies can provide insight into in situ barriers and enablers to participation specific to the intervention, population and setting [13, 14], including those related to study design (e.g. eligibility, participation requirements), participants (e.g. willingness, language and communication needs), practitioners (e.g. clinical and research role imbalances), ethics (e.g. privacy, risk to individual patients), practice (e.g. space, staffing), collaboration (e.g. interprofessional engagement, co-location of research and clinical staff) and health systems (e.g. policies) [49, 50].

This paper reports findings from a qualitative sub-study nested within medicinal cannabis trials for symptom burden relief in patients with advanced cancer. The primary aim was to identify enablers and barriers to recruitment and participation from patients’ perspectives, informing the refinement of current and future RCTs.

### Trial context

Following 2016 legislation permitting limited access to cannabis for research and medicinal purposes in Australia [51], a pilot study (MedCan Pilot) [52], followed by MedCan 1 [42] and 2 [53], was established to generate an evidence-base for cannabis use in palliative care [43, 54–56]. Patients eligible for inclusion had advanced cancer (metastatic or locally advanced) with associated symptom burden as measured by an Edmonton Symptom Assessment Scale (ESAS) total symptom distress score (TSDS) of ≥10 for cancer-related symptoms and at least one individual ESAS score ≥3. All participants were required to abstain from consuming other cannabis products and, for MedCan Pilot and MedCan 2, refrain from driving while on the trial. Those consenting to be part of the study continued to receive standard palliative care (see Table 1 and published protocols).

**Table 1:** MedCan study details

	MedCan Pilot	MedCan 1	MedCan 2
Objective	To investigate the feasibility of drawing on global symptom burden scales to assess response to cannabidiol (CBD) or tetrahydrocannabinol (THC) To determine median tolerated doses of CBD and THC To document adverse events	To assess the effect of escalating doses of CBD versus placebo in the management of symptom burden in patients with advanced cancer	To investigate the safety and efficacy of escalating doses of CBD/THC versus placebo for the management of symptom burden in patients with advanced cancer
Design	Prospective two-arm open-label pilot trial	Multi-centre, blinded randomised placebo controlled trial	Multi-centre, blinded, randomised, placebo-controlled trial
Trial interventions	Arm 1: escalating doses of CBD delivered orally via an oil. Arm 2: escalating doses of THC delivered orally via an oil.	Arm 1: Intervention: escalating doses of CBD delivered orally via an oil. Two-week patient-determined titration phase to reach symptom burden relief with tolerable side effects, followed by an optional 2 weeks of a stable dose. Arm 2: Placebo: matching placebo oil solution.	Arm 1: Intervention: escalating doses of THC and CBD delivered in a 1:1 ratio orally via an oil. Two-week patient-determined titration phase to reach symptom burden relief with tolerable side effects, followed by 2 weeks of a stable dose. Arm 2: Placebo: matching placebo oil solution.
Primary outcomes	Number of participants screened and number of participants completing 14 and 28 days	Change in TSDS from baseline to day 14	Change in TSDS from baseline to day 14
Trial status and registration	Complete: ACTRN12618001205224 Registered July 2018 (MedCan – Pilot)	Complete (under analysis): ACTRN12618001220257 Registered July 2018 (MedCan 1 – CBD)	Ongoing: ACTRN12619000037101 Registered January 2019 (MedCan – 2 THC/CBD)
Ethical approval	HREC/18/MHS/83	HREC/18/MHS/43; HREC 18/16	HREC/MML/49348; HREC 18/36
Funder	NHMRC MRFF Grant APP1152232	NHMRC MRFF Grant APP1140160	

## Methods

### Study design

Anticipating recruitment challenges [2, 5], a nested qualitative sub-study (TalkingMedCan) was undertaken in 2019–2020 with patients who had either met eligibility criteria to participate in MedCan Pilot, 1 or 2 (MedCan) and enrolled to participate in a trial, or who were eligible to participate but declined. Semi-structured interviews were selected for data collection for their capacity to offer inductive insight, while accommodating the time and communication needs of patients with advanced cancer [57, 58]. Ethical approval for TalkingMedCan was obtained from the Human Research Ethics Committees at the hospitals providing care (HREC/17/MHS/97;HREC 17/27).

### Sampling and recruitment

A purposive strategy was adopted to gain a balanced sample based on gender, age and diagnosis and to obtain maximum variation. Recruitment was led by trial investigators and research nurses who would identify eligible patients. A copy of the participant information sheet and consent form was provided, facilitating a verbal discussion with the patient on the risks and benefits of participation. Once a patient agreed to participate and provide written consent, the clinical trial coordinator liaised with the patient and research team to arrange a suitable time and place for the interview.

### Participants

Forty-eight patients with advanced cancer undertook semi-structured interviews for TalkingMedCan (*n*=28 who consented to participate in a trial and *n*=20 who declined). Of those who consented, most were participating in MedCan1 (*n*=2 MedCan Pilot, *n*=23 MedCan1, *n*=3 MedCan2). Aiming for sampling variation, the most common primary cancer diagnoses were breast, prostate and lung (Table 2); others included bladder, bile duct, colorectal, endometrial, gastro-oesophageal, glioma, kidney, mesothelioma, ovarian, pancreatic, rectal and urothelial. The majority of participants were receiving anticancer therapy. Only 8 (17%) of the 48 patients interviewed reported no comorbidities.

**Table 2:** Demographic characteristics of interview participants

Characteristic	Interview participants		
	Trial participant (***n*** = 28)	Declined trial participation (***n*** = 20)	Total (***n*** = 48)
Gender, % (*n*)			
Male	25% (12)	22.91% (11)	47.91% (23)
Female	33.33% (16)	18.75% (9)	52.08% (25)
Age in years, % (*n*)			
≤49	6.25% (3)		6.25% (3)
50–69	31.25% (15)	20.8% (9)	50% (24)
70–89	20.83% (10)	22.91% (11)	43.75% (21)
Household arrangements, % (*n*)			
Living with others (e.g. spouse, children)	54.17% (26)	31.25% (15)	85.42% (41)
Living alone	4.16% (2)	10.42% (5)	14.58% (7)
Ethnicity, % (*n*)			
Anglo-Saxon/English	35.41% (17)	37.5% (18)	72.92% (35)
Australian	6.25% (3)		6.25% (3)
Pacific Islander	6.25% (3)		6.35% (3)
Australasian	2.08% (1)		2.08% (1)
Scottish	2.08% (1)		2.08% (1)
Undisclosed	6.25% (3)	1.16% (2)	10.41% (5)
Education level, % (*n*)			
Did not complete high school	6.25% (3)		6.25% (3)
High school	50% (24)	33.33% (16)	83.33% (40)
Bachelor’s degree		2.08% (1)	2.08% (1)
Unknown	2.08% (1)	6.25% (3)	8.33% (4)
Primary cancer diagnosis, % (*n*)			
Breast	12.5% (6)	12.5% (6)	25% (12)
Prostate	6.25% (3)	14.58% (7)	20.83% (10)
Lung	8.33% (4)	6.25% (3)	14.58% (7)
Ovarian	6.25% (3)		6.25% (3)
Endometrial	6.25% (3)		6.25% (3)
Urothelial		4.16% (2)	4.16% (2)
Colorectal/rectal	4.16% (2)		4.16% (2)
Pancreatic	4.16% (2)		4.16% (2)
Other	10.42% (5)	4.16% (2)	14.58% (7)

Most participants were aged >50 years and identified as female of Anglo-Saxon or English ethnicity. Some identified as Australian or Pacific Islander. Twelve/23 male participants, and 16/25 female participants had enrolled in a clinical trial at the time of interview; 9/25 female participants and 11/23 male participants declined trial participation. Forty-one participants were living in a household with others (most commonly their spouse only, *n*=26). All but 8 participants reported secondary school as their highest level of education.

### Semi-structured interviews

All interviews—lasting between 20 and 60 min—were facilitated by a member of the research team not involved in patient care with interviewing experience and recorded using a digital voice recorder before being transcribed verbatim. Most interviews were conducted in a private hospital consultation room, directly following an appointment at the hospital (*n*=42). After the onset of COVID-19, and in accordance with an approved ethics amendment, six interviews were conducted via telephone. Family/support members were permitted to be present in interviews. Several accepted this invitation, but any verbal contributions to interviews were omitted from final analysis. Interview questions were designed to foster in-depth exploration of patients’ perceptions of medicinal cannabis, and of barriers and enablers to participation in MedCan trials (Table 3). Aligned with iterative approaches to data collection, data generated in earlier interviews informed refinements to the semi-structured interview guide (e.g. prompts regarding financial and transport considerations). This iterative approach also enabled identification of data saturation during analysis [44, 59].

**Table 3:** Interview guide

1. Tell me about your cancer journey.	
2. Tell me your views about cannabis generally; tell me your views on medicinal cannabis specifically.	
3. What informed your views on cannabis and medicinal cannabis?	
4. What has been your experience with cannabis in the past?	
5. Are you aware of current laws on medicinal cannabis in Australia?	
6. What are your main reasons for participating/not participating in the trial?	
7. Do you have any concerns about medicinal cannabis use?	
8. What do you view as the potential benefits or harm of medicinal cannabis use?	
9. Do you think medicinal cannabis should be available in the future? If yes, how should it be made available?	

### Data analysis

Transcripts were analysed thematically and, by case, prompting us to consider patterns across and the context within the data, and guided by inductive and iterative approaches to interpretivist thematic analysis and constructivist grounded theory [60, 61]. Analysis was led by an experienced qualitative health researcher, with advanced training in the social sciences. Triangulation, including multiple team members with backgrounds in sociology, anthropology and medicine, involved three team members (RO, PG, TD) closely reading five transcripts and discussing themes prior to formal coding, and then three team members (AS, RO, MD) reviewing data displays and coding frameworks in the latter stages of analysis. This fostered insight into study implications and their relevance to differing audiences. NVivo 12 qualitative analysis software—with files saved to a password-protected data management platform—was used to support data management, and to create and organise codes based on the research aims, interview questions, and emerging findings. Participant details (e.g. name, address) were stored in a separate location to transcripts and NVivo files to prevent unnecessary cross-referencing. Inductive, deductive and abductive techniques were employed [61–63]. Open coding was undertaken to identify new themes and facilitate cross-data source comparison. In the latter stages, themes were mapped against the literature [7, 49].

Recruitment and analysis ended at the point of theoretical saturation, when no new themes were seen to emerge and data was sufficient for pattern recognition across purposively sampled groups [59]. This paper describes and explores the interrelated key themes of enablers and barriers to trial participation, from patients’ perspectives. Other findings on participants’ perceptions of medicinal cannabis are reported elsewhere [64].

## Results

In this section, we present findings of why interviewees accepted or declined invitations to participate in MedCan using summary tables. De-identified quotes are presented with reference to each interviewee’s participant number, gender (F/M) and decision regarding RCT participation. Aligned with constructivist approaches to thematic analysis [60, 62], we explicitly reference scholarship informing deductive elements of analysis, highlighting which findings are novel to the intervention, context and population.

### Enablers of trial participation

Despite some of the widely reported challenges to recruitment in RCTs generally, and within palliative care contexts more specifically [5], interest in participating in the MedCan trials (Pilot, MedCan1, MedCan2) was strong. Illustrating the enthusiasm for the study, one interviewee described their interest as ‘a little bit of altruism… a lot of curiosity’ (P30M, declined). Others, after hearing ‘so many good things that are happening with it [medicinal cannabis]’ (P10F, agreed), wanted to ‘give it a go’ (P21F, agreed), saying ‘Let me try it’ (P7M, agreed).

Key enablers to trial participation are shown in Tables 4, 5, 6, and 7 aligned with previous research showing perceived benefits to self and others to be central [7, 9, 11]. Anticipated benefits, however, were often specific to medicinal cannabis. Possible self-benefits encompassed specific symptom control, generalised wellbeing and a sense of doing everything in one’s capacity to manage one’s quality of life, a belief (however indirect) in cannabis’s curative potential and a hope for viable and more ‘natural’ alternatives to current or previous medications. Perceived potential benefits to others from participation were wide ranging: contributions to knowledge, possible cure for cancer, and political efforts to legalise cannabis.

**Table 4:** Enablers: perceived benefits to self, I

Theme	Sub-theme	Description	Data displays
Symptom burden relief	Pain relief	A desire for relief from current and anticipated pain associated with advanced cancer, treatment and comorbidities	My main reason is to help me with the pain… so I can maybe do things. (P7M, agreed)
			I’m hoping the cannabis oil will help me with my pain, if I get any pain. (P9M, agreed)
	Relief from other symptoms	Anticipated relief from symptoms of fatigue, nausea and low appetite	My fatigue has been so bad for quite a while now…if this is going to help with the pain and help with fatigue then why not give it a go? (P21F, agreed)
			Sometimes appetite and sometimes fatigue. But the pain is the main thing. (P19F, agreed)
	Exhausting all options	A desire to explore all opportunities for symptom burden relief	Let me try it, let me do it. I want all the help I can get. (P7M, agreed)
			Try anything if I think it’s going to help… I’ve got to try something. (P16M, agreed)
Improved general wellbeing		Improved wellbeing was a motivation for others who anticipated benefiting through improved physical functioning, joy, coping and hope	I want to be able to walk, go for my 6 km walk….Play golf…. I can’t do that anymore…. it’d be nice if this stuff [medicinal cannabis] can… get rid of all my aches and pains so that when I have got life on this earth it’s a good happy life. (P22M, agreed)
			The main reason I’m participating in the trial is to maybe - it being helpful with me coping. (P23F, agreed)
			Just a bit of hope really. Anything what’s going to be a help. It’s a bonus…. if it helps me a tiny bit I’m – you know, I’m grateful. (P24M, agreed)

**Table 5:** Enablers: perceived benefits to self, II

Theme	Sub-theme	Description	Data displays
Anti-cancer effect	Alluded hope	A hope, that medicinal cannabis would have an effect on their cancer and/or prolong their life	I want to see if it’s going to help me... I hope it can help me, my cancer. Or at least how I deal with it. (P10F, agreed)
			I’ll try anything. They’re looking around for another something now to see if there’s a trial for me. I’m on it. I’m desperate. I want to live. I don’t want to die. (P13F, agreed)
			Whatever’s going to prolong my life, I’m going to give it a crack, and I think that’s human nature isn’t it. (P14F, agreed)
	Directly stated hope	Hope that medicinal cannabis would have an effect on the cancer and/or prolong life	I’m told it won’t help the cancer, but who knows? It might. (P6F, agreed)
			It’s all through my bones so I’ll try anything to try and fix it up. (P16M, agreed)
			I want something to work for me. I want it to control the disease for me. It cannot – it won’t cure it but so long as it controls it. Prolong my lifespan. That’s all I want. (P26F, agreed)

**Table 6:** Enablers: perceived benefits to self, III

Theme	Sub-theme	Description	Data displays
Alternative	Natural	A desire for interventions perceived to be more natural	I’m really interested in [a] more natural way of treating my body alongside the fact that I do have to put poisons and toxins in my body (P8F, agreed)
	Substitution	A desire to decrease one’s intake of pharmaceutical interventions	I really, really wanted to cut back on a lot of the medications I’m currently taking. If I can just have this and my chemo tablets, I’d be happy… I want to come off, wean myself off them…. to help me come off at least, at least 70 per cent of my oral medication. (P25F, agreed)
	Fewer/less intense side-effects	Wanting to improve quality of life through reducing their intake of other interventions with undesired side-effects	I’ve turned down another drug because of its side effects. I would rather have cannabis that has no side effects. (P10F, agreed)
			Just in the hope that it was an easier way of relieving pain while I’m on this journey. Most pain killers either send you to the loo or don’t - one or the other extremes. Maybe that [medicinal cannabis] would be more gentle - I don’t know. (P12M, agreed)

**Table 7:** Enablers: perceived benefits to others

Theme	Sub-theme	Description	Data displays
Altruism	Broad	Helping others—in addition to or instead of themselves—as a primary impetus in their MedCan participation decision	I will go on any trial if somewhere along the line it will help you, help your friends or help somebody else, and you’ve got to have trials to find all this stuff out. (P22M, agreed)
			Well [my participation in MedCan is] certainly not self-interest because you’ve got no guarantee that… you’re not an active recipient in the trial. You could be on a placebo…. So, it’s about just the greater good of the research… hopefully in some small way it can help. (P47M, agreed)
	Anti-cancer	Altruism combined with a hope that medicinal cannabis had anti-cancer effects	I realise my fight is nearly over, but as I said I’ll willingly take any trial that might increase the possibility of a - of the next person surviving. That’s the only reason I’m doing it. (P11M, agreed)
	Legalisation	Altruism underpinned by political enthusiasm for cannabis legalisation	One of my reasons [for participating in MedCan] is I hope it can help the cause to legalise it. (P10F, agreed)

#### Perceived benefits to self

##### Symptom burden relief and general wellbeing

Many patients spoke of their experiences of pain and anticipation of further pain, both treatment- and disease-related, as well as pain related to comorbid conditions. Potential benefit to nausea and appetite was another common perceived benefit. Patients were explicit in wanting ‘to try anything’ (P2F, agreed; P13F, agreed; P16M, agreed; see Table 4 Sub-theme ‘exhausting all options’) to address symptoms and side-effects: ‘grasping at straws… to see if it eases my way forward’ (P4F, agreed). For others, interest in participating in MedCan was propelled by expectations of ameliorated capacity, and improved wellbeing through better physical and emotional functioning, such as an ability to ‘walk….play golf’ (P22M, agreed) or a generalised improved ‘coping’ capacity (P23F, agreed; see Table 4 Theme ‘Improved general wellbeing’).

##### Belief in anti-cancer effect

Some patients were partially motivated by perceived anti-cancer effects. For these participants, discussions of exhausting all options for symptom relief and exhausting all options for treating disease progression blurred together, raising questions about participants’ understanding of the trial’s purpose. Interviewee P10F (agreed), for example, described her ‘hope it can help me, my cancer. Or at least how I deal with it’ (see Table 5, Sub-theme ‘Alluded hope’). Other participants more candidly indicated a belief in the anti-cancer effect of medicinal cannabis, often alongside acknowledgement and comprehension that the clinical trial was not designed with this in mind. Interviewee P6F (agreed), for instance, stated, ‘I'm told it won't help the cancer, but who knows? It might’ (see Table 5, Sub-theme ‘Directly stated hope’).

##### Cannabis as an alternative to other medications

For many interviewees, medicinal cannabis was described as a preferable, more ‘natural’ (P10F, agreed) or ‘herbal’ (P14F, agreed) alternative to other medications for symptom management, prompting patients to consider participation in the trial (see Table 6, Sub-theme ‘Natural’). Such perceptions may reflect patients’ relative lack of consultation with health professionals and limited knowledge of cannabis (medicinal or recreational). Only 2 participants described consulting a health care professional outside of the trial team to inform their decision making. Others saw medicinal cannabis as a ‘gentle’ (P12M, agreed) alternative to current pharmaceutical interventions that were considered to have undesirable side-effects (see Table 6, Sub-theme ‘Fewer/less intense side-effects’) or saw MedCan as a possible way to ‘cut back’ (P25F, agreed) their in-take of medications overall (see Table 6, Sub-theme ‘Substitution’).

#### Perceived benefit to others: altruism as a driver for trial participation

Aligned with previous scholarship on RCT recruitment [7, 9], altruism, or benefits to others, was another common driver behind patients’ decisions to participate (see Table 7, Sub-theme ‘Broad’). For some, such altruism was combined with a belief that such a benefit would include testing of medicinal cannabis’ anti-cancer properties, that the trial would improve ‘the possibility of…the next person surviving’ (P11M, agreed; see Table 7, Sub-theme ‘Anti-cancer’). For others, it was combined with a hope that the trial would inform efforts to further de-criminalise and legalise marijuana in Australia (see Table 7, Sub-theme ‘Legalisation’).

### Barriers to participation

Here we report barriers to trial participation. Self-selection was evident; some patients considered their symptom burden insufficient to warrant participation. Highlighting which findings are unique to MedCan, we first present barriers known to be common to RCTs. Subsequently, we present concerns specific to the population, study design and research setting: intersecting health and logistical challenges, restrictions on driving, fears of hallucinatory side-effects, cost and preferences for cannabis sourced outside of the clinical trial.

#### Common barriers

Here we first present barriers common to RCTs: wanting to avoid randomisation, fear of unknown side-effects and interactions with current medications, a preference to maintain one’s current pharmaceutical schedule, gatekeeper protection and unproven benefits to self [7, 49, 58, 65, 66]. Several interviewees declined participation due to randomisation, preferring to know if they were receiving a placebo or an intervention (see Table 8, Theme ‘Randomisation’). One went as far as asking, ‘Why should I be a guinea pig for someone else to find out whether it’s going to be any good for everyone else or not?’ (P25M, declined), contrasting the altruistic motivations described above and alluding to an aversion to the positioning of subjects in RCTs. For other interviewees, trial participation decision-making hinged on an assessment of current symptom burden, available symptom management options and the relative benefit of medicinal cannabis, or lack thereof (See Table 8, Theme ‘Uncertain benefit to self’). In weighing up benefits to self and risks [8], these interviewees saw the risk of possible side-effects and risk of unwanted interaction with their current medications as outweighing the benefits—particularly with the benefits not yet proven, and when current symptom burden was perceived to be low or sufficiently controlled (see Table 8, Sub-themes ‘Fear of side-effects’ and ‘Fear of interactions’). One interviewee expressed an extreme concern that the interaction of medicinal cannabis with his other medication could be fatal and cited advice from his general practitioner that medicinal cannabis was unlikely to be beneficial (see Table 8, Sub-themes ‘Fear of fatal interactions’ and ‘Clinician advice’).

**Table 8:** Barriers: common reasons for declining RCT participation

Theme	Sub-theme	Description	Data displays
Randomisation		Preference to know if they were receiving an intervention	I couldn’t see me coming in every week and having to - and plus the fact that I might not get it. I might get it; I might not get it. (P31F, declined)
			I said, ‘well, I’m not interested because I don’t want to take the chance of having a placebo.’ So that was that. (P32M, declined)
Risks	Fear of side-effects	Risks of further side-effects from medicinal cannabis outweighing possible benefits	I was keen on [trial participation]… but then I found out the after - the side effects that can come with it and I think, ‘oh, I go through enough side effects right now.…’ that sort of scared me a bit. (P45F, declined)
	Fear of interactions	Concerns that medicinal cannabis would interact with other medications	I’m on things that really are working well… and I don’t want to upset the apple cart… I’m going so well on what I’ve got on and I’m taking, I don’t see the point of having anything extra. (P43F, declined)
	Fear of fatal interactions	Concern that medicinal cannabis might interact with other medications, with possibly fatal consequences	I don’t think I’ll take it on mate. I just don’t want to take the chance in case it’s - it might affect my, all my medications what I take and it might kill me. (P35M, declined)
Gatekeeper	Clinician advice	Advice from a clinician that medicinal cannabis would not be beneficial	What I’ve been told about it by people like a GP…. He says, no, don’t take it because it probably wouldn’t do you any good. (P35M, declined)
Uncertain benefit to self	Unproven	Intervention’s benefit yet to be rigorously established	It’s in the unproven basket still. That’s my only concern, unproven. (P36M, declined)
	Not enough symptom burden to benefit	Self-assessed symptom burden too low to reap any perceived benefits	I have no pain as such so I can’t see the need for me to have it as yet or to go on the trial… Well if I haven’t got any pain at the moment there’s not much point taking it is there. (P33F, declined)
	Symptom burden managed—no perceived benefit to self	Pain, nausea and fatigue were being adequately managed	I don’t think it’s worth starting now…. I am in a lot of pain but I’m managing – it’s being managed with patches. (P27F, declined)
			I’m not having as much issue with the symptoms at the moment… the pain and the nausea…. I wasn’t eating much and I was losing weight, but I’ve got my appetite back again. (P42F, declined)

#### Intersecting health and logistical concerns

As has been identified previously in trials in which potential participant cohorts may include patients who are older, frailer and/or face specific socio-economic challenges [49, 65, 67–69], the logistics of trial participation proved a significant barrier for many interviewees. MedCan study design required participants to attend weekly hospital appointments and access prescriptions (medicinal cannabis or placebo) via the hospital pharmacy. Several patients cited their current physical health and related limitations in their capacity to travel to attend regular hospital appointments as factors in their decision to decline trial participation. Interviewee P44M (declined), for example, described his ‘exercise tolerance as abysmal’, preventing him from traversing the 100 m from the bus stop to the hospital necessary to attend weekly appointments (see Table 9, Sub-theme ‘Physical health’). These health challenges often intersected with and compounded other challenges: required travel time, cost and inconvenience of public transport exacerbating time poverty and financial concerns (see Table 9, Sub-themes ‘Transport’, ‘Time’ and ‘Distance’). One interviewee stated: ‘I couldn’t see me coming in every week…. We live not that far away, but we come in on the train and it’s a long time. I’m just so tired and everything from the cancer that it just - yeah… [And] if I come in the car, it’s expensive’ (P16F, declined).

**Table 9:** Intersecting barriers: physical health and study-design logistics

Theme	Sub-theme	Description	Data displays
Weekly hospital appointments	Physical health	Poor health/stamina and inhibited capacity to walk or travel to attend hospital appointments	[I]f it had been a year and a half to two years ago, I would have made sure I got in to do it. I’d have been happy to do the trial… But the state I’m in now, I’m just not able. (P27F, declined)
			My exercise tolerance is abysmal; I walk 10 metres and I’m stuffed… I would be willing to be involved in a trial, if it only consisted of taking a dose of medication once a day … and reporting by phone…to… climb up that bloody hill…[from the train station to the hospital] is becoming more and more - I mean, my wife said, the last time, ‘it looks like I’m going to have to get you a wheelchair’ (P44M, declined)
	Transport	Lack of consistent transportation	It was with coming out and in all the time. I’ve got a good half hour’s journey and I don’t always have someone to bring me in. So, it’s a bit of a problem getting transport in. That’s the only reason [for my not participating in the trial]. (P27F, declined)
	Time	Time commitment associated with weekly hospital appointments seen as too burdensome by participants with work and competing specialist appointments	When Dr [de-identified] said, I’ll have to come in here every week, ‘I’m going oh, I have enough trouble coming in here once a month…’ I do casual work every week and that’s just too much…. If I had to come in every week for the marijuana, no, it would be all too much. (P28M, declined)
			I chose not to go onto the study, there’s enough on my plate to be quite honest without getting involved…. with another commitment… (P40M, declined)
	Distance	Time and financial costs deemed too onerous by those living far from the hospital	I heard about it and I was all for it. It just really - we couldn’t fit it in to our time slots. We’re from the country, it takes us three and a half hours to get down here. It would have cost us out of pocket a fair bit of money for accommodation, travel, et cetera. (P37M, declined)

#### Concerns regarding medicinal cannabis

Several barriers to participation specific to medicinal cannabis emerged during analysis. Most prominent, the trial requirement for MedCan Pilot and 2 (both of which involve THC) to discontinue driving featured heavily in decision-making for some interviewees (see Table 10, Theme ‘Driving restrictions’). A few interviewees identified concerns about medicinal cannabis and/or marijuana more generally, such as a fear that medicinal cannabis would cause them to experience hallucinations or a high (see Table 10, Theme ‘Fear of getting “high”’). Though access to medicinal cannabis (or placebo) was free to participants while on MedCan, cost of future access to medicinal cannabis posed a barrier to some (see Table 10, Theme ‘Cost’). A trial requirement that participants cease current cannabis use prior to enrolling in the trial, and the related risk of being randomised to the placebo arm, featured as barriers for a minority (see Table 10, Theme ‘Staying on Cannabis’). For one interviewee, a decision to decline trial participation was based on a perception that the cannabis they acquire informally has fewer additives than the medicinal cannabis available through MedCan (see Table 10, Theme ‘Preference for unaltered cannabis’).

**Table 10:** Barriers: specific to cannabis

Theme	Description	Data displays
Driving restriction	Requirement that MedCan participants abstain from driving	The only thing that’s stopping me is not being able to drive… Public transport is something that I’ve never used, ever and most times where I’ve lived it’s just not convenient. (P42F, declined)
		To be stuck home not driving and it’d just kill the quality of life. The only thing that helps keep my sanity these days is I play a bit of golf. If I can’t get there, well, I would just be sitting and moping around at home. (P39M, declined)
Fear of getting ‘high’	Perceived risk of experiencing an altered state	I’m a logical thinking person. Cannabis is going to have me off with the fairies…. I don’t know that, if it’s going to have me off with the fairies. I’m not going to have a full life if I’m off in the clouds somewhere. (P39M, declined)
Cost	Prohibitive cost of medicinal cannabis following the trial	I was told that once it got - if it got legalised and all the rest of it, it was going to be $300 or $400 a [pop] …. that’s out of the reach for a lot of people. Why are they even doing it if we’re not even going to be able to afford to have it? (P31F, declined)
Staying on cannabis	Risk of being assigned to the placebo arm	I know that half the people in it will be on placebos. I don’t think I can afford to be off the [non-trial] cannabis... Once you’re on it it’s very important that you stay on it, especially if you’ve got ongoing problems. That’s why I wouldn’t want to be part of the [trial]. If I was to have it and know it was cannabis I’d be happy to be monitored but as far as having the risk of having a placebo, there’s no point in that. (P38F, declined)
Preference for unaltered cannabis	Perception that community acquired cannabis was purer	I know of some people who have done research and they said there’s a couple of little nasties in [the medicinal cannabis]... Where I’m getting it from, their base is totally coconut oil, so it’s not having any little nasties in it. Don’t ask me about the nasties because I don’t understand. (P46F, declined)

## Discussion

TalkingMedCan offers novel insight into perceptions of medicinal cannabis from patients’ perspectives in an Australian palliative care setting, and related enablers of and barriers to recruitment. Overall, findings show the merits of nesting qualitative sub-studies into RCTs, to uncover in situ recruitment challenges related to the setting, intervention and study population. The insights garnered supported revisions made to subsequent RCT study designs, with implications for medicinal cannabis RCT research more broadly.

Our study found curiosity in the intervention, perceived self-benefit and altruism to be central MedCan participation enablers. Participants were motivated by a desire to manage their symptoms using a ‘natural’ treatment with few expected adverse effects. Others have similarly identified perceived benefits to self and others [7, 9–11], along with patient enthusiasm, as participant enablers—adding to the limited but growing trend of scholarship reporting enablers to RCT participation, not just barriers [49]. Interest in medicinal cannabis specifically, as a milder ‘herbal’ intervention, likely reflects the growing aversion to opioids [70] and increasing interest in cannabis and its legalisation in Australia [41, 43].

Many interviewees described a desire to exhaust all options in symptom burden relief as a reason for taking part in the trial, particularly if conventional options were deemed insufficient or problematic. In some interviewees, discussions of exhausting all options for symptom burden relief blurred with exhausting all options for a cure or life-prolonging interventions—a common motivation behind oncology RCT participation [66]. This finding, however, raises questions aligned with research in other oncology populations [10, 71], of potential participants’ understanding of the trial intervention, and how this understanding relates to participation decision-making and intentions following the trial.

There was little discordance between perspectives on trial participation found in this study and perspectives on medicinal cannabis more broadly in Australia, with studies suggesting a general acceptance of and lack of concern regarding medicinal cannabis for treatment of physical and mental health conditions [40, 41], including use amongst cancer patients [28]. A minority of interviewees held expectations—ranging from dim to direct hope—that medicinal cannabis could be curative, again aligned with existing Australian studies [43].

Key barriers underpinning decisions to decline participation in MedCan included gatekeeper influence, perceptions of risks outweighing benefits and aversion to randomised trials of unproven interventions. These reflect and confirm well-established barriers within the literature on RCT participation decision-making [8, 13, 49, 58, 66]. Specifically, when symptom burden was being sufficiently managed, several participants expressed a fear that trial participation would ‘upset the apple cart’—adversely interacting with their careful regime of medications. Others self-assessed their own health status, symptom burden and consequent potential to benefit and declined to participate because of uncertain benefit.

The most prominent barriers related to the study requirements, its setting and legal restrictions. MedCan participants were asked to regularly attend appointments at the hospital to assess changes in symptom burden and fill their prescriptions, and aligned with current laws, patients enrolled in MedCan Pilot and 2 were not permitted to drive a car. Low stamina and difficulty obtaining transportation led to some declining to participate. Team et al. [49] describe such difficulties as study-related and participant-related barriers; frailty and fatigue preventing participation are common in RCTs with older participants [58, 65]. This finding also highlighted setting-specific considerations, regarding the city’s transport system, and supported a recommended revision to our study design. In a subsequent RCT, patients are given the option to attend appointments related to trial participation in person or via telephone appointments. The number of hospitals and hospital pharmacies involved has also been extended to include regional and rural hospitals, decreasing the travel burden associated with participation. Such revisions are necessary to accommodate potential future COVID-19 public health restrictions, while also responding to in situ needs of study participants.

Our study also identified barriers specific to medicinal cannabis: concerns about experiencing a substance-induced high, future cost and preferences to maintain their use of recreational cannabis for medicinal purposes. While these findings support research showing concerns related to medicinal cannabis are relatively minimal in Australia [43], they also indicate that such concerns are distinct and worthy of consideration. In contrast to research conducted in North America and Israel [30, 35], stigma did not appear to be a prominent concern amongst interviewees. These finding suggest that barriers to RCT participation are both common *and* context-specific [49]; studies are always embedded within hospital, cultural and urban systems, with distinct policies, legal-political structures, urban density or sprawl and associated public transportation systems. Thus, while some have called for a ‘methodological shift’ toward employing the existing ‘wealth of knowledge’ on RCT recruitment challenges, rather than accumulating further evidence [13], this study shows the value of nesting qualitative sub-studies into RCTs: identifying and countering intervention- and setting-specific hurdles [14].

### Insights for medicinal cannabis RCTs

Interest in medicinal cannabis is high [26], driven by strong public interest and word of mouth [35]. It is noteworthy that few interviewees in this study described seeking or receiving information about medicinal cannabis from a health care professional, instead more often opting to obtain information and opinions from popular media, family, and friends. As such, suggestions within the RCT recruitment literature that focus on increasing awareness of the health problem and of the benefits of the intervention [7, 9] are to some degree already attended to within the context of RCTs of medicinal cannabis in advanced cancer care settings—patients have a high level of awareness of the intervention. Health professionals, however, need a stronger evidence base to guide patients with confidence [26], and this warrants further RCTs into medicinal cannabis in palliative and other care settings.

Tailored insights are needed to address specific recruitment and design challenges in this setting. Here, we offer suggestions for others designing RCTs and recruiting patients with advanced cancer to medicinal cannabis RCTs.

- Foreground patients’ and families’ strong desires for hope [72–74]. In communication with patients prior to initiating consent, check that prospective participants understand the aim of the intervention, specifically that medicinal cannabis is unlikely to be curative.
- Emphasise the potential for findings to inform policy and practice, providing evidence for legislative decision making.
- Consider the research setting. Holding follow-up appointments via telephone or at community healthcare centres [49] and facilitating dispensary at local pharmacies may make the RCT more accessible to those prohibited from driving or who would otherwise struggle with significant travel requirements. Alternatively, taxi vouchers may help to lessen the financial, support and fatigue challenges associated with hospital visits [49].

## Study strengths and limitations

Our study is the first to explore advanced cancer patients’ perspectives on enablers and barriers to participation in an Australian medicinal cannabis RCT for symptom burden relief. Although previous research contributes important understanding of common facilitators and impediments to RCT participation [7, 8, 49] as well as Australian patients’ perceptions of medicinal cannabis [28, 43], this study offers findings specific to both this population and intervention. A further strength is the inclusion of not only eligible patients who agreed to participate in the RCT, but also those who declined.

A limitation of this study could be the relatively homogeneous sample, with limited cultural, socio-economic, age and geographical diversity. Few participants described cultural backgrounds outside of the majority population in Australia; none identified as Aboriginal or Torres Strait Islander. Most participants described completing secondary school; few had achieved post-secondary qualifications.

## Conclusion

TalkingMedCan provided insight into enablers and barriers to recruitment, supporting revisions to subsequent study designs. Findings offer novel insight into recruitment considerations specific to this population and intervention, and corroborate research showing concerns regarding cannabis are minimal and distinct in Australia, with a minority of patients holding curative expectations or concerns about serious side-effects. Future research should explore how the revisions to study design suggested here—foregrounding hope, emphasising the potential for RCTs to lead to policy change, and allowing participation via telehealth—change enablers and barriers to recruitment. Overall, findings support calls for a context-sensitive approach to RCT design, suggesting the merits of embedding qualitative sub-studies into RCTs to identify recruitment challenges that may be unique to the intervention, setting and population.

## Data Availability

Data generated and analysed for the current study are available to suitably qualified individuals by request, from the corresponding author, subject to HREC approval.
